# 21 Lower Extremity Burns in Low- and Middle-Income Countries: Analysis of the WHO Global Burn Registry

**DOI:** 10.1093/jbcr/iraf019.021

**Published:** 2025-04-01

**Authors:** Daniel Najafali, Megan Najafali, José Arellano, Hilary Liu, Saeid Rezaei, Logan Galbraith, Erik Reiche, Quincy Tran, Victor Stams, Francesco Egro

**Affiliations:** Carle Illinois College of Medicine; Loyola University Chicago Stritch School of Medicine; University of Pittsburgh Medical Center; University of Pittsburgh Medical Center; University of Pittsburgh Medical Center; Northeast Ohio Medical University; University of Pittsburgh Medical Center; University of Maryland School of Medicine; Carle Foundation Hospital; University of Pittsburgh Medical Center

## Abstract

**Introduction:**

Burns involving the lower extremities can severely impact mobility and significantly reduce quality of life. There is a dearth of information on lower extremity burns in low-resource settings, particularly in low- and middle-income countries (LMIC). In high-income countries, conditions like diabetes complicate the management of these injuries and affect recovery potential. We aim to characterize lower extremity burns and evaluate their impact on outcomes on a global scale. It was hypothesized that lower extremity burns are an independent risk factor for mortality and, given amputation rates in high-income countries post-burn, are mostly managed surgically.

**Methods:**

The WHO Global Burn Registry were analyzed from inception to September 2024. Individuals from high-income countries were excluded. Descriptive analyses were used to stratify patients with lower extremity burns and those without lower extremity burns. Multivariable logistic regressions assessed predictors with the primary outcome of mortality. Secondary outcome measures were surgical intervention and functional impairment at discharge for survivors.

**Results:**

There were 2,752 individuals who had a lower extremity burn managed in LMICs, representing 66% of total cases (N=4,169). Compared to non-lower extremity burns, individuals with lower extremity burn injuries were older (median ages: 27 years vs. 18 years, P< 0.001), mostly males (51%), consisted of flame injuries (63%), had higher TBSA, more instances of inhalation injury, and had significantly higher Baux and modified Baux Scores (P< 0.001). Most lower extremity burn patients did not undergo surgery (60%) and most were not discharged (51%). The mortality rate for lower extremity burns was 38% compared to 12% for non-lower extremity burns (P< 0.001). There were increased odds of mortality with lower extremity burns (OR 1.67, 95%CI 1.33-2.09) and they led to a significantly higher likelihood of surgical intervention (OR 1.49, 95%CI 1.29-1.73) in multivariable analysis.

**Conclusions:**

Lower extremity burns in LMICs predisposed patients to require surgical intervention and higher mortality. Understanding the degree of amputations in LMICs will better capture the impact these injuries have on long-term function and quality of life.

**Applicability of Research to Practice:**

Assessing amputation rates and the availability of prosthetics and rehabilitation services in LMICs is essential for improving long-term outcomes and quality of life. The results could guide policy changes and resource allocation by global health organizations and local governments to address these disparities and improve burn care in low-resource settings.

**Funding for the Study:**

N/A

